# The Book World of Medicine and Science

**Published:** 1904-03-19

**Authors:** 


### The Book World of Medicine and Science.

How to Keep Well: an Explanation of Modern Methods of Preventing Disease. By Floyd M. Crandall, M.D. (London: Grant Richards. 1904. Pp. 511. Price 6s.)

The author of this volume addresses himself rather to the educated layman than to the medical practitioner, to whom indeed much of his matter is necessarily familiar; although some of the later portions are admirable in their suggestiveness. Starting with the causes of disease, especially those of a microbic nature, he sketches the chief infectious diseases met with in this country and in America with special relation to the way in which they spread and the precautions which can most readily be taken against them. The production of artificial immunity by the use of antitoxins is then popularly treated, most space as is natural being devoted to that of diphtheria. The mode of preparation of antidiphtheritic serum as well as its beneficial results in the reduction of the mortality from diphtheria are stated in such a way as to be readily intelligible. Vaccination receives a chapter to itself in which this important subject is treated with moderation and fairness. The statistics, which are well brought up to date, leave little room for doubt in any unbiassed mind as to the advantages of vaccination. The effects of modern life on disease, heredity, and the rearing of children are likewise discussed in a practical manner, and here, as in the subsequent chapters on the dangers of middle life and the prevention of break down, the oft-repeated caution against the evils of over-pressure, whether in business or pleasure, is again insisted on and moderation advised with practical learning and sound common sense. The conditions and diseases of sedentary and advanced life are suitably treated, and the writer adds a chapter on modern surgery in which the influences of asepsis and anæsthesia are duly emphasised. It is not to be expected that such a volume will convert the anti-medical "faddist," but whether used by individuals as a guide to health or considered as a popular exposition of what preventive medicine has done and what it seeks to accomplish, the influence of the book will be to further a belief in scientific methods as opposed to quackery and ignorance, and so to make for progress.

Charles White, F.R.S.: A Great Provincial Surgeon and Obstetrician of the Eighteenth Century. By Charles J. Cullingworth, M.D., F.R.C.P. (London: Henry J. Glaisher. 1904. 8vo. Pp. viii. and 56. With 4 illustrations. Price 2s. 6d. net.)

Dr. Cullingworth has done good service to medical biography and to the medical school at Manchester by printing the address on Charles White which he delivered before the Medical Society of Manchester on October 7th, 1903. Charles White's personality and influence were very great. He was in touch scientifically with John Hunter and his pupils; in practice he is renowned for his book on the Management of Pregnant and Lying-in Women, wherein he taught that there is a close analogy between the fever that followed surgical operation and the fever to which lying-in women are liable; as a citizen he was influential in founding the Manchester Literary and Philosophical Society, and a College of Arts and Sciences. But he was also a surgeon of no mean repute, and to the literary public of to-day he is known by De Quincy's references to his museum. Dr. Cullingworth has produced a very complete memoir of this great man, and his researches have enabled him, not only to clear up several points in his career which have hitherto been doubtful, but they have enabled him to draw attention to the portrait of Charles White by J. Allen, the Lancashire artist. This fine picture was long believed to have been lost irretrievably, but it is in the possession of Mr. J. C. Swan, of Cullercoats, and Dr. Cullingworth has reproduced Ward's engraving of it as a frontispiece to his book. The other illustrations give interesting pictures of old Manchester. They represent Charles White's house in King Street, the Manchester Infirmary, and Lunatic Hospital.

Aids to Surgery. By Joseph Cunning, M.B., B.S., F.R.C.S.Eng. (London: Baillière, Tindall and Cox. Pp. 394. Price 4s. 6d.)

The author has intended this book for students preparing for examination, and the manner in which it is arranged or this purpose is admirable. To any student of sufficient intelligence to perceive that he cannot learn surgery from a text-book of this kind, a brief and yet comprehensive summary such as this will prove highly serviceable. By glancing through its pages he can open up afresh the channels of knowledge that he has already gained, and so arrange his ideas as to facilitate their expression whether under the stress of examination or of actual practice. We can strongly recommend Mr. Cunning's book to those who will use it in the manner thus indicated.

Herbert Fry's Royal Guide to the London Charities. Edited by John Lane. (London: Chatto and Windus. 1904. Price 1s. 6d.)

This well-known guide contains a large amount of information bearing on the various London charities, including the objects for which the individual institutions were founded, the names of their chief officials, and details of how, when, and where applications for aid or information are to be made. It should prove of service in providing the poor and needy with the means of learning where and how they may seek to obtain relief.

The Local Government Annual and Official Directory, 1904. Edited by S. Edgecumbe Rogers. ("The Local Government Journal" Office, 27a Farringdon Street, E.C. Price 1s. 6d.)

This publication constitutes a very complete and well-arranged book of reference for matters connected with local government, giving the names and addresses of the chief officials of the various county and borough councils, boards of guardians, county and borough asylums, etc., throughout the kingdom. In addition to this directory there is given a quantity of information respecting such matters as open spaces, public baths and washhouses, and much else that comes under local government administration. Specially useful are the sections which deal with local government legislation during 1903, and the constitution of the Metropolitan Water Board, and the various education committees in England and Wales.

Who's Who, 1904. (London: Messrs. Adam and Charles Black. Price 7s. 6d. net.)

This most indispensable reference book grows apace, than which there can be no better sign of its popularity. In glancing through the 1,700 pages which the volume comprises it is impossible to avoid being struck by the generosity which has been shown in selecting the individuals whose names are thought worthy of mention. However, if a fault at all, this is at least one on the right side.

Who's Who Year-book. (Price 1s. net.)

This excellent little volume consists in the main of various tables, which include, for example, lists of Government Officials, Fellows of the Royal Society, Societies and their Secretaries, Peculiarly Pronounced Proper Names, Principal Schools, Railways, and Steamship Lines, and a vast deal of information of a similar kind, which is often very much desired but not readily obtained. The book supplies a distinct want, and we hope to see it established as a recognised annual.

